# Impact of, Factors for the Success of, and Concerns Regarding Transplant Patients’ Skin Cancer Campaigns: Observational Study

**DOI:** 10.2196/43845

**Published:** 2023-06-14

**Authors:** Erica Mark, Joseph Nguyen, Fatima Choudhary, Jules B Lipoff

**Affiliations:** 1 School of Medicine University of Virginia Charlottesville, VA United States; 2 Advanced Dermatology and Cosmetic Surgery Philadelphia, PA United States

**Keywords:** GoFundMe, transplant, skin cancer, nonmelanoma skin cancer, crowdfunding, fundraising, crowdsourcing, insurance, demographic, squamous cell carcinoma, basal cell carcinoma, multivariate linear regression, binary logistic regression

## Abstract

**Background:**

Due to rising health care costs, patients have sought alternative ways of addressing medical expenses. In particular, transplant patients have complex and expensive medical needs—including skin cancer surveillance—that may not be fully covered by insurance. One such method of financing medical costs is by crowdsourcing through web-based platforms, most notably GoFundMe.

**Objective:**

Previous work identified factors associated with GoFundMe campaigns’ fundraising success for dermatologic diseases. We sought to characterize these factors in transplant recipients’ campaigns for funds raised for covering skin cancer–related costs. These factors include demographics, campaign traits, and subjective themes.

**Methods:**

From January to April 2022, we analyzed GoFundMe campaigns using the following search terms chosen on the basis of author consensus: “transplant skin cancer,” “transplant basal cell,” “transplant squamous,” “transplant melanoma,” and “dermatologist transplant.” Demographic data were coded from campaign text or subjectively coded based on author consensus. Campaigns were read completely by 2 independent coders and associated with up to 3 different themes. Linear regression was performed to examine the qualities associated with success, which was defined as funds raised when controlling for campaign goals. Logistic regression was used to examine qualities associated with extremely successful campaigns, defined as those raising funds over 1.5 times the IQR.

**Results:**

Across 82 campaigns, we identified several factors that were associated with fundraiser success. Patients who experienced complications during infectious disease treatment, those who received a pancreas transplant, or those who died from their disease raised significantly more money. Patients older than 61 years raised significantly less money. Extremely successful campaigns (>US $20,177) were associated with campaigners who emphasized a disability from their disease, those who were reluctant to ask for help, or those who died due to their disease.

**Conclusions:**

Demographic and thematic factors are associated with transplant patients’ skin cancer–related fundraising success, favoring those who are younger, in more extreme situations, and appear reluctant to ask for help; these findings are consistent with those of previous studies. Additionally, transplant patients have complex and expensive dermatologic needs that may not be fully covered by insurance, as reflected in their GoFundMe campaigns. The most commonly mentioned reasons for fundraising included living expenses or loss of income, inadequate or no insurance, and end-of-life costs. Our findings may inform transplant patients how to maximize the success of their campaigns and highlight gaps in health care coverage for skin cancer–related costs. Limitations include the possibility for misclassification due to the data abstraction process and limiting data collection to fundraisers available on GoFundMe while excluding those on other websites. Further research should investigate the ethical implications of crowdfunding, financial needs of this patient population, and potential ways to improve access to routine skin cancer surveillance among patients receiving transplants.

## Introduction

GoFundMe is a web-based crowdfunding platform used to raise money for individual campaigns. Though not originally designed to raise medical funds, as of 2021, one-third of all funds raised on GoFundMe (US $650 million) are designated for covering medical costs [1]. These fundraisers aim to raise money to cover both the medical and nonmedical costs of disease, such as income support. Campaigns seek support for a wide range of medical conditions, including those for patients with a history of organ or bone marrow transplantation seeking funds for needs related to their long-term care.

Transplant patients often require costly and time-intensive multidisciplinary care. While taking immunosuppressive medication, transplant patients are encouraged to establish care with a dermatologist for regular skin cancer screenings as they are 20-100 times more likely to develop skin cancers [2]. Squamous and basal cell carcinoma comprise 90% of skin cancers among transplant recipients, with risk increasing over the duration of immunosuppressive therapy: a 65-250-fold increase in risk in squamous cell carcinoma and a 10-fold increase in risk in basal cell carcinoma [3].

Patients post organ transplantation represent a unique population with needs for regular screening, preventative therapies, and, if needed, skin cancer treatment. Our group has previously characterized the broad range of dermatologic GoFundMe campaigns and analyzed factors associated with fundraising success [4]. Given established skin cancer risks post transplantation secondary to immunosuppression [3], in this study, we aimed to characterize and analyze these factors in transplant recipients’ campaigns for skin cancer–related fundraising.

## Methods

This study was deemed exempt by the University of Virginia’s institutional review board. From January to April 2022, we analyzed all available GoFundMe campaigns, created between 2013 and 2021, using the following search terms chosen through author consensus: “transplant skin cancer,” “transplant basal cell,” “transplant squamous,” “transplant melanoma,” and “dermatologist transplant.” Exclusion criteria included campaigns active for less than a day or if organ transplantation occurred after the diagnosis of skin cancer. Demographic data were coded from the campaign’s text or subjectively coded based on author consensus. Qualitative themes were coded until thematic saturation was reached, using an inductive qualitative method [5]. Campaigns were read completely by 2 independent coders and associated with up to 3 different themes.

The cleaned data were exported to R (version 4.0.2; The R Foundation). The frequencies of the themes were calculated based on the percentage of times the theme was mentioned. Two separate models were used due to concerns regarding collinearity. Multivariate linear regression was performed to investigate the amount of funds raised against demographic and thematic variables. The IQR method for identifying outliers was applied to the amount of funds raised and the goal of the campaign. Based on this outlier detection method, campaigns that raised more than US $20,177 were excluded from this regression analysis. Binary logistic regression was performed to compare demographic variables and themes among fundraisers that raised above US $20,177 to those that raised below this amount in order to investigate qualities associated with extreme success in fundraising. Extreme success was defined as an amount over 1.5 times the IQR (>US $20,177). The significance threshold was set at *P*<.05.

## Results

As shown in Table 1, the majority of campaign recipients were male (n=59, 72%), older than 61 years (n=43, 52.4%), and White (n=79, 96.3%). Only a minority of campaigns were created by the recipients themselves (n=10, 12.2%), with most campaigns created by a family member (n=35, 42.7%) or friend (n=32, 39.0%). Though most campaigners did not specify the type of skin cancer, among those who did, the majority were related to melanoma (n=19, 45.2%) and squamous cell carcinoma (n=17, 40.5%). The following transplant subtypes were seen most often: kidney (n=35, 42.7%), bone marrow (n=16, 19.5%), lung (n=16, 19.5%), heart (n=10, 12.2%), liver (n=8, 9.8%), and pancreas (n=6, 7.3%). The campaigns raised an average of US $7656 and had an average goal of US $16,072 (Table 2). Few campaigns met their goal (n=22, 26.8%). The most commonly mentioned themes included the cost of daily living or inability to work, inadequate or no insurance, and end-of-life costs (Table 1).

With respect to demographic characteristics, campaigns for patients older than 61 years earned an average of US $6983 less than those for patients aged 21-40 years (*P*=.009). Those who mentioned that their treatment was complicated by an infection (US $7512, *P*=.008) or those who ultimately died from their disease (US $7946, *P*<.001) raised significantly more funds. Lastly, those who had a pancreas transplant raised more funds than those who did not (US $6878, *P*=.04; Table 3). With respect to extreme positive outliers (raising >US $20,177), those who mentioned that the campaign recipient was disabled by their disease (odds ratio [OR] 1.139, 95% CI 1.006-1.312), who were those reluctant to ask for help (OR 0.184, 95% CI 1.012-1.427), or those who died from their condition (OR 0.167, 95% CI 1.045-1.336) were associated with extreme success (Table 3).

**Table 1:** Demographics and themes of transplant patients’ skin cancer GoFundMe campaigns (N=82).

Characteristics and themes			Values, n (%)
**Gender**			
	Female	23 (28)	
	Male	59 (72)	
**Age^a^ (years)**			
	11-20	2 (2.4)	
	21-40	14 (17.1)	
	41-60	23 (28)	
	≥61	43 (52.4)	
**Race^b^**			
	White	79 (96.3)	
	Hispanic	2 (2.4)	
	African American	1 (1.2)	
**Type of skin cancer**			
	Unspecified	40 (48.8)	
	Melanoma	19 (23.2)	
	Squamous cell carcinoma	17 (20.7)	
	Basal cell carcinoma	6 (7.3)	
**Relationship to the creator of the campaign**			
	Self	10 (12.2)	
	Partner	5 (6.1)	
	Family member	35 (42.7)	
	Friend	32 (39)	
**Number of skin cancers**			
	1	44 (53.7)	
	≥2	38 (46.3)	
**Had a liver transplant**			
	No	74 (90.2)	
	Yes	8 (9.8)	
**Had a kidney transplant**			
	No	47 (57.3)	
	Yes	35 (42.7)	
**Had a lung transplant**			
	No	66 (80.5)	
	Yes	16 (19.5)	
**Had a heart transplant**			
	No	72 (87.8)	
	Yes	10 (12.2)	
**Had a bone marrow transplant**			
	No	66 (80.5)	
	Yes	16 (19.5)	
**Had a pancreas transplant**			
	No	76 (92.7)	
	Yes	6 (7.3)	
**Used Immunosuppressives**			
	Not Mentioned	31 (37.8)	
	Yes	51 (62.2)	
**Died from cancer**			
	No	61 (74.4)	
	Yes	21 (25.6)	
**Seen by a dermatologist**			
	No	75 (91.4)	
	Yes	7 (8.5)	
**Top 5 most common themes for fundraising^c^**			
	Cost of living	46 (25)	
	Inadequate insurance	34 (18.5)	
	End-of-life costs	24 (13)	
	Travel costs	22 (12)	
	Inability to work	16 (8.7)	

^a^Age could only be evaluated as a categorical variable as many fundraisers referenced the decade of life but not specific ages.

^b^Race was either explicitly mentioned in the campaign or subjectively assigned based on author consensus.

^c^Themes (n=184) were coded through an inductive qualitative method until thematic saturation was reached, meaning that themes were continuously added as they appeared in the data until no novel themes emerged. Each campaign was read completely by 2 independent coders and was associated with any discernible themes.

**Table 2:** Fundraising metrics of transplant patients’ skin cancer GoFundMe campaigns.

	Values	
	Mean (SD)	Median
Amount raised (US $)	7655.89 (9002.40)	3902.50
Goal of the fundraiser (US $)	16,071.94 (19,871.52)	10,000
Number of donors	73.55 (76.23)	43
Number of updates	3.54 (6.61)	2

**Table 3:** Stepwise linear (I)^a^ and logistic (II)^b^ regression analyses of demographic and thematic variables against the amount raised^c^.

									β (SE)			Odds ratio (95% CI)			*P* value
**Stepwise linear (I) regression; dependent variable: amount raised**															
	**Age group (years; reference: 21-40 years)**														
					11-20			–5074 (6226)			N/A^d^			.42	
					41-60			–4171 (2440)			N/A			.09	
					≥61			–6983 (2609)^e^			N/A			.009	
	**Gender (reference: female)**														
				Male				1017 (1931)			N/A			.60	
	**Fundraiser themes**														
			Multiple comorbidities					–3142 (2340)			N/A			.18	
			Infection complicating treatment					7512 (2788)^e^			N/A			.009	
			Psychosocial					–5959 (3089)			N/A			.06	
	**Miscellaneous^f^**														
						≥2 cancers	2612 (1763)			N/A			.14		
						Seen by a dermatologist	4634 (3085)			N/A			.14		
						Kidney transplant	–3396 (1805)			N/A			.06		
						Pancreas transplant	6878 (3357)^g^			N/A			.04		
						Immunosuppressives used	–3075 (1758)			N/A			.09		
						Insurance	6135 (4222)			N/A			.15		
						Died from cancer	7946 (1954)^h^			N/A			<.001		
**Logistic (II) regressions with outliers with themes as variables**															
	Goal							0.000005949 (0.000001445)^h^			1.000 (1.000-1.000)			*<.*001	
	**Age group (years; reference: 21-40 years)**														
		11-20						0.02029 (0.1831)			1.021 (0.713-1.461)			.91	
		41-60						–0.04783 (0.07381)			0.953 (0.825-1.102)			.52	
		≥61						–0.07517 (0.08198)			0.928 (0.790-1.089)			.36	
	**Gender (reference: female)**														
		Male						0.08243 (0.06081)			1.086 (0.964-1.223)			.18	
	**Fundraiser themes**														
		Disability due to disease						0.1391 (0.06768)^g^			1.149 (1.006-1.312)			.04	
		Reluctance to ask for help						0.1838 (0.08760)^g^			1.202 (1.012-1.427)			.04	
	**Miscellaneous**														
		Time of fundraiser						–0.00003655 (0.00003554)			1.000 (1.000-1.000)			.31	
		Died from cancer						0.1667 (0.06279)^e^			1.181 (1.045-1.336)			.01	

^a^Regression I is a linear regression that depicts demographic and thematic variables against the dependent variable: the total amount raised.

^b^Regression II is a binary logistic regression investigating demographic and thematic variables more likely to be seen in extremely successful campaigns compared to the majority of campaigns.

^c^Campaigns were defined as extremely successful if they raised more than 1.5 times the IQR (ie, greater than US $20,177).

^d^N/A: not applicable.

^e^*P*<.01.

^f^Adjusted *R*^2^=0.3457

^g^*P*<.05.

^h^*P*<001.

## Discussion

### Principal Findings

Transplant patients have complex and expensive dermatologic needs that may not be fully covered by insurance, as reflected in their GoFundMe campaigns [3,6]. In our qualitative study, the most commonly mentioned reasons for fundraising included living expenses or loss of income, inadequate or no insurance, and end-of-life costs. Our findings corroborate known gaps in transplant care, namely the difficulty for patients to afford basic necessities due to a loss of income or inadequate coverage, challenges in accessing care, and long-term care and rehabilitation needs [6]. Additionally, we found that most skin cancer–afflicted transplant campaigners seeking help were White men older than 61 years, and that specific demographic and campaign characteristics were associated with fundraiser success.

As of 2022, most transplant recipients were male (62%), White (52%), and older than 50 years (62%) [7]. While it is expected that White transplant patients become afflicted with skin cancers at higher rates than patients of color, the percentage of non-White campaigners in this study was very low (3/82, 3.7%) [3]. Additionally, 46.3% of campaigners in this study had more than 1 skin cancer and a majority of fundraisers (n=75, 91.4%) did not mention dermatologist visits prior to the development of skin cancers. It is possible that transplant patients without routine dermatologic surveillance are more likely to experience extreme presentations of skin cancer, thus requiring crowdfunding. While the lack of mention in the campaign does not preclude the possibility of prior dermatology visits, it may suggest a lower likelihood of well-established or sustained care. Additionally, in our study, several patients died (11/82, 13%) from skin cancer, transplant complications, or other diseases, exemplifying the need for improved transplant skin cancer–related follow-up and coverage. Overall, our data support the development of more robust skin cancer education for this patient population.

When analyzing factors associated with success, our findings are consistent with those of previous studies that reported that successful campaigns often feature extreme stories, emphasizing an inability to work or hesitancy to ask for help [2,8]. As mentioned above, those aged ≤60 years also found significantly greater success, which is congruent with our group’s findings in a prior study [4] where the most successful fundraisers included patients aged 20-40 years. We hypothesize that fundraiser crowd appeal may in part depend on an ability to demonstrate supposed deservingness and garner sympathy from potential contributors. As mentioned above, a number of themes were associated with fundraiser success. Inability to work, disability from disease, and infectious complications suggest a debilitating and urgent condition, and reluctance to ask for help may portray a picture of resilience following the exhaustion of other means of fundraising, both of which may garner sympathy from potential donors. This finding on crowdsourcing for skin cancer following transplantation parallels the results from crowdsourcing for all cancers, where campaigns for high-mortality cancers that used emotional words to prompt empathy had raised more funds [9]. In another of our group’s previous studies, where we analyzed factors associated with fundraiser success in GoFundMe campaigns raising money for plastic surgery conditions, we found that themes including inadequate insurance, travel costs, life-saving treatment, and end-of-life expenses were associated with a higher amount of funds raised (E Mark et al, unpublished data, May 2022). Similarly, these themes may suggest resilience (traveling far to receive care) and urgency (inadequate insurance or life-saving treatment). Interestingly, end-of-life expenses in our prior study and death from disease in this study were associated with greater success, suggesting that reverence for life may garner sympathy from potential donors. In summary, themes suggesting resilience, severity or urgency, and mortality are associated with greater success. As campaigns increase, competition may favor those who can create compelling narratives, adding to the disparities that health care providers must discern.

### Limitations

Limitations include the possibility for misclassification due to the data abstraction process, limiting analysis to fundraisers on GoFundMe while excluding those on other websites, and inability to distinguish between anonymous donations and those from friends or family members. Campaign textual features, such as clarity, grammar, and complexity, were difficult to quantify in this study but may affect fundraiser success. Additionally, due to the limited information available on GoFundMe, factors such as education attainment, income, social support, cultural background, and differences in health systems that could impact fundraiser success could not be analyzed. Another limitation is the lack of generalizability of our findings. The results of this study are largely based on campaigns for White men older than 61 years and may not fully represent the outcomes of individuals with different cultural or socioeconomic backgrounds. Further, while this study included global campaigns, the results were heavily skewed to reflect crowdsourcing in the US health care system, which does not provide universal care like other high-income countries. Differences in health care coverage would likely impact the need for individual fundraising, thereby making these results less generalizable across health care systems.

### Conclusions

In summary, demographic and thematic factors are associated with the success of transplant patients’ skin cancer–related fundraising campaigns, favoring those who are younger, in more extreme situations, and appear reluctant to ask for help. Further research should investigate the ethical implications of crowdfunding, the financial needs of this patient population, and potential ways to improve access to routine skin cancer surveillance among patients receiving transplants.

## Abbreviations

OR: odds ratio
